# Training the proprioceptive and propulsive function of the foot for improved technique and enhanced performance of the golf swing: a pilot study

**DOI:** 10.3389/fspor.2026.1888684

**Published:** 2026-09-14

**Authors:** Angelo Belotti, Niccolò Bisazza, Andrea Calcari, Paolo Perrone, Roberto Panichi, Andrea Biscarini

**Affiliations:** 1 Department of Medicine and Surgery, University of Perugia, Perugia, Italy; 2 Federazione Italiana Golf, Rome, Italy; 3 Federazione Italiana Golf – Comitato Lombardo, Milano, Italy

**Keywords:** ball speed, club speed, golf, ground reaction, proprioception, swing

## Abstract

**Background:**

The proprioceptive and propulsive functions of the feet—despite their essential role in stability, movement initiation, and efficient bottom-up energy transfer along the kinetic chain—are largely overlooked in conventional golf physical and technical training. To address this gap, we developed a novel approach to golf athletic training and swing technique that embeds foot mechanics as a fundamental driver of performance enhancement.

**Methods:**

Twenty-five experienced golf players completed three separate swing sessions: the first before a specific foot-proprioceptive intervention (time *t*_0_), the second immediately after the intervention (time *t*_1_), and the third three minutes after the end of the second session (time *t*_2_). In the second and third sessions, participants were instructed to modify their swing technique by integrating the sequence of foot actions practiced during the proprioceptive intervention. Club and ball kinematics and vertical ground-reaction force (vGR) under each foot were recorded and compared across sessions.

**Results:**

Lead-foot peak vGR increased from *t*_0_ to *t*_1_ but did not increase further from *t*_1_ to *t*_2_. Club speed increased from *t*_0_ to *t*_1_ and continued to rise, albeit to a lesser extent, from *t*_1_ to *t*_2_. Ball speed did not change significantly from *t*_0_ to *t*_1_ but increased from *t*_1_ to *t*_2_. The observed changes following the foot-focused intervention and the corresponding modifications to the swing technique suggested a progressive propagation, with practice, from the feet to the club and, ultimately, to the ball.

**Conclusion:**

These acute findings suggest that the proposed exercises may facilitate a progressive refinement of swing technique through greater integration of foot function, consistent with the observed improvements in swing-performance variables.

## Introduction

Golf has become increasingly popular in recent decades and is now widely practiced across recreational and competitive levels, yet technical considerations still tend to receive more attention than athletic preparation (1–4). Mastery of swing mechanics, precision, and consistency has traditionally been prioritized, placing the golf instructor at the center of player development from youth programs to the professional level and sustaining the longstanding view of golf as a predominantly skill-based sport (5–8). Only in recent years, with a growing understanding of the physical demands and determinants of the game, has strength and conditioning emerged as an integral component of young athletes' and elite players' practice (9–15).

In golf-specific athletic training, alongside general muscular strength and power exercises, there is strong emphasis on training leg-hip, trunk power and grip strength (3, 6, 16). However, this often overlooks the proprioceptive and propulsive functions of the feet, which are fundamental for initiating and controlling movements, providing stability, and generating propulsive force (17–20). In other sports involving complex, coordinated ground-based actions, training programs place greater importance on the role of the feet (19). In golf, this oversight may lead to suboptimal performance and increased injury risk, as foundational support and movement efficiency can be partially compromised (21).

In contrast, in many other sports, the integration of athletic preparation with technical training is standard practice. From the outset, athletes are trained with a focus on multilateral development and the conditioning of individual physical and coordinative capacities (22). This comprehensive approach is often recommended to precede the progressive learning of sport-specific technical skills (23, 24). In athletics, the role of the feet has been extensively studied and recognized as crucial for performance, fine motor control, and joint stability throughout the lower-limb kinetic chain (25). Consequently, specific exercises aimed at enhancing foot function are integral to training across all track-and-field disciplines (26).

The golf swing shares key biomechanical features with throwing motions in athletics, such as javelin, shot put, and baseball pitching. These disciplines demonstrate the effectiveness of a progressive bottom-up timing sequence, in which movements are initiated from the ground with the action of the feet and propagate upwards through the kinetic chain (27). This sequential activation facilitates efficient transfer of energy from the lower to the upper extremities, maximizing endpoint velocity at release and enhancing performance while reducing injury risk (28–30). In golf, applying this principle may help maximize clubhead speed and promote a more efficient swing, highlighting the critical role of foot mechanics in performance (31).

Golf-specific studies have provided increasing evidence of the importance of foot–ground interaction during the swing. Han et al. (32) identified significant associations between ground-reaction-force parameters, particularly those of the lead foot, and maximum clubhead speed in highly skilled golfers. Faux et al. (33) further showed that experimentally altering foot center of pressure can modify lower-body kinematics during the golf swing, while a recent systematic review confirmed the relevance of ground-reaction-force and center-of-pressure variables to clubhead speed and skill level (34). However, previous studies have primarily characterized golfer–ground interactions or experimentally manipulated foot center of pressure. To our knowledge, this is the first study to develop and experimentally evaluate a structured, golf-specific foot-conditioning protocol designed for immediate integration into swing technique, while concurrently characterizing acute changes in lead-foot peak vertical ground-reaction force (vGR), club speed, and ball speed in highly skilled golfers.

The above literature review highlights that the proprioceptive and propulsive functions of the feet—despite their critical role in providing stability, initiating movement, and ensuring efficient energy transfer along the kinetic chain—are largely overlooked in traditional golf training. Recognizing this gap, this study aimed to introduce a novel approach to golf athletic training and swing technique that emphasizes foot mechanics as a fundamental component of performance. Specifically, we developed a proprioceptive exercise sequence targeting foot mechanics, characterized by a rhythmic, sequential pattern of intended forefoot-pressing and horizontal shear actions, accompanied by alternating loading of the rearfoot and forefoot regions of both the lead and trail feet. After completing this conditioning phase, experienced golfers were instructed to perform successive swing sessions on professional golf courses, integrating the learned motor sequence into their swing technique. We hypothesized that the intervention and the subsequent application of the practiced foot-action sequence during the swing sessions would be associated with a progressive short-term adjustment of swing technique and corresponding changes in performance-related variables. More specifically, we hypothesized that the key performance-related swing parameters—namely, lead-foot peak vGR, club speed, and ball speed—would progressively increase during the swing sessions performed with the modified swing technique acquired during the conditioning intervention.

The results of this study could offer valuable insights into the development of young golfers and the optimization of athletic preparation and performance for professionals. The integration of foot-focused exercises into golf training could help bridge the gap between technical mastery and athletic capacity, leading to enhanced performance.

## Materials and methods

### Participants

Twenty-five healthy male golf players were recruited from Italian golf clubs. Participant characteristics relevant to the study are summarized in Table 1. To obtain a relatively homogeneous sample in terms of sex, training background, competitive experience, and familiarity with instrumented swing assessment, participants were considered eligible if they were male, aged between 18 and 65 years, currently active in golf, had at least 5 consecutive years of competitive experience, and regularly practiced golf for a minimum of 2 h per week. Previous experience with a launch monitor was also required, including prior assessment of ball speed. Participants were excluded if they reported a current musculoskeletal injury, a history of pathological conditions affecting the trunk or upper or lower limbs, including the foot and ankle, that could interfere with swing execution, or an inability to perform the golf swing pain-free and with appropriate form and technique. An *a priori* sample-size calculation was performed before participant recruitment using G*Power version 3.1.9.7 for the planned within-subject repeated-measures ANOVA. Assuming *α* = 0.05, a statistical power of 80%, and an effect size *f*(*V*) of 0.65 using Cohen's convention (35), with one group and three repeated measurements, the resulting target sample size was 25 participants. This sample size was also consistent with those commonly adopted in comparable golf studies (10). The study was conducted in accordance with the Declaration of Helsinki and was approved by the Ethics Committee of the University of Perugia (protocol No. 226211). All participants provided written informed consent for their inclusion in the study prior to participation.

**Table 1:** Participant characteristics relevant to the study.

Characteristic	Value
Number of participants	25
Age (mean ± SD)	29 ± 10 years
Height (mean ± SD)	181 ± 8 cm
Body mass (mean ± SD)	78 ± 9 kg
Years of golf experience (mean ± SD)	20 ± 10 years
Weekly hours of golf practice (mean ± SD)	16 ± 12 h
Handicap index (mean ± SD)	0.4 ± 2.7
Percentage of participants downswing from right to left	100%
Percentage of participants using glasses	40%

### Proprioceptive intervention

All participants underwent a single specific foot proprioceptive intervention lasting 3 min. Each player assumed his usual starting swing position. From this position, the feet performed a rhythmic sequence of actions, structured in four phases. For golfers with a downswing from right to left, the action begins with the participant pressing the left forefoot into the ground while performing an intended horizontal shear action directed laterally against the ground, initiating a bottom-up chain of movement in the left lower limb—eversion of the rear foot joints (subtalar and transverse tarsal joints), knee flexion, and hip external rotation—followed by right pelvic rotation (phase 1). The left foot action also causes a partial shift of the body weight towards the right rear foot, promoting rear foot inversion, partial knee extension, and internal hip rotation in the right lower limb, with further right pelvic rotation (phase 2). The action then proceeds in the opposite direction in the next two phases. The participant then presses the right forefoot into the ground while performing an intended horizontal shear action directed laterally against the ground, promoting rearfoot eversion and hip external rotation in the right lower limb and changing pelvic rotation from right to left (phase 3). This right foot action simultaneously shifts the body weight towards the left rear foot, with rear foot inversion, knee extension, and hip internal rotation in the left lower limb, promoting further left pelvic rotation (phase 4). During the entire sequence, the exerciser was instructed to avoid lateral trunk tilt, and strive to maintain knee alignment limiting varus/valgus and internal/external rotations, thus protecting both the knee and lower back joints. Although no golfers with a downswing from left to right participated in this study (as shown in Table 1), it is worth noting that, for such golfers, the actions would be reversed, starting with the right forefoot.

The sequence of actions was first thoroughly explained to the participants by a professional trainer, then practically demonstrated, and finally practiced under the trainer's supervision. All interventions were instructed and supervised by the same professional trainer, who used the same explanation, demonstration, and verbal-cueing procedure for every participant. Because the intervention was actively performed rather than externally applied, the intended forefoot-pressing and horizontal shear actions were generated voluntarily by each participant. No absolute force target was prescribed; participants were instructed to perform the prescribed actions at their maximal voluntary effort while maintaining the prescribed movement pattern, joint alignment, and postural control. In the first minute of conditioning, the four phases were performed at a very slow pace to become familiar with the exercise, gradually acquiring the movement pattern. In the second minute of conditioning, the four phases were performed with a smoother and faster rhythm to consolidate and then automate the pattern, creating a continuous sequence. In the third and final minute, the sequence was performed by each golfer with the timing of their own swing. Throughout the intervention, a 5-s pause was taken between each complete cycle (four phases). This exercise progression was designed to facilitate the immediate voluntary integration of the practiced foot-action sequence into the swing.

The duration of the 3-min foot proprioceptive conditioning intervention was considered sufficient for enabling experienced golfers to voluntarily integrate the practiced foot motor sequence into their swing technique during swing sessions conducted immediately after the intervention (refer to the following “Training and testing sessions” section). Extending the intervention duration could potentially induce fatigue, which might negatively impact performance in these sessions.

### Training and testing sessions

The proprioceptive intervention and the testing swing sessions were conducted at the “Golf della Montecchia” course (27 holes, Par 72, 6,318 meters; Selvazzano dentro Padova, Italy) and the “Golf Club Perugia” course (18 holes, Par 71, 5,763 meters; Perugia, Italy). Participants were asked to perform their usual pre-competition warm-up routine, which was supplemented by inviting each golfer to freely execute swing shots monitored by a launch monitor (see *Data Recording and Processing* section), until achieving ball speed values they perceived as stable and representative of their typical performance. This procedure helped ensure a consistent performance baseline prior to the first testing session. After the warm-up, participants performed three separate testing swing sessions, each consisting of five swing shots using a 6-iron golf club: the first session occurred before the foot proprioceptive intervention (time *t*_0_), the second soon after the intervention (time *t*_1_), and the third three minutes after the end of the second session (time *t*_2_). During the second and third sessions, participants were instructed to change their swing technique by integrating the sequence of foot actions practiced during the proprioceptive intervention into their swing. In the three minutes between the second and third sessions, participants were asked to focus on the sensations experienced in the second session with the new movement technique. In each session, participants were free to choose the rest period (typically less than 1 min) between one shot and the next.

### Data recording and processing

The speed of the club head at ball impact (club speed) and the initial ball speed after the impact (ball speed) were recorded during the three swing sessions using the markerless Trackman launch monitor (Vedbæk, Denmark), which is equipped with synchronized high-speed (4,600 fps) dual cameras and 24 GHz dual Doppler radar technology. Notably, two recent research studies have demonstrated excellent within- and between-session reliability for both club head speed and ball speed measurements obtained using the Trackman launch monitor (36, 37).

A Smar*t2*Move 1D Dual Force Plate system (Allschwil, Switzerland), specifically designed for golf force analysis, was used to record independent left- and right-foot vGR and centers of pressure. The system is based on force gauges, transmits force data in real time at 120 Hz, and has a vertical-force measurement range of 100–2,350 N. According to the manufacturer, its performance and reliability have been validated through independent university biomechanics testing (38). Before each recorded trial, the force plates were calibrated according to the standardized procedure prescribed by the manufacturer, including unloaded and participant-loaded calibration steps. A time-synchronized camera captured high-resolution images at 240 fps. The native software of data analysis considers 7 reference positions (P1–P7) during the swing action (see Appendix). The Smar*t2*Move Dual Force Plate system can be synchronized with TrackMan data to assess changes in vGR associated with the modified swing technique and their relationships with club and ball speed.

In each session and for each participant, the five values of each parameter measured across the five swing shots were averaged, and the resulting mean values were then used in the statistical analysis.

### Statistical analysis

An ANOVA design was employed to analyze the data samples. The ANOVA assumptions of sphericity, normality, and homogeneity of variance were verified with the tests of Mauchly, Shapiro–Wilk, and Levene, respectively. When necessary to meet these assumptions, probabilities were estimated using the Greenhouse–Geisser and Huynh–Feldt corrections, and/or data were transformed with the natural logarithm (ln) function. Descriptive statistics, presented as mean ± SD, consistently refer to the untransformed data, even if the analysis was performed on transformed data. The primary inferential analysis of TrackMan and force-plate data was a one-way repeated-measures ANOVA, with time [before (*t*_0_), immediately after (*t*_1_), and three minutes after (*t*_2_) the proprioceptive training intervention] as a three-level within-subject factor. *Post hoc* pairwise comparisons were performed using the Scheffé procedure, which controls the family-wise Type I error rate.

In addition to calculating the average performance at different stages of the treatment (*t*_0_, *t*_1_, and *t*_2_) for the entire group, it was also of interest to calculate the individual differences between the performances at two different stages of the treatment for each athlete, average these differences over the participants, and perform one-sample *t*-tests on these sets of data (null hypothesis mean = 0, alternative hypothesis mean ≠ 0). These difference-score analyses were considered secondary and complementary and were used to describe the direction and magnitude of the individual within-subject changes; the primary inferential conclusions were based on the repeated-measures ANOVA and Scheffé *post hoc* comparisons.

The statistical power was evaluated using the observed power (ω), while the effect size was measured by partial eta squared $\left(\eta_{p}^{2}\right)$ for ANOVA tests and Cohen's *d* for *t*-tests. Values of $\eta_{p}^{2}$ of 0.01, 0.06, and 0.14 and Cohen's *d* values of 0.2, 0.5, and 0.8 were interpreted as small, medium, and large effects, respectively (35). For all statistical analyses, the significance level was set at *p* < 0.05. The Origin software package was used for statistical calculations.

## Results

Repeated-measures ANOVA revealed a significant effect of time on lead-foot peak vGR $\left(\;p<0.001,\omega >0.99,\eta_{p}^{2}=0.35\right)$, club speed $\left(\;p<0.001,\omega =1.00,\eta_{p}^{2}=0.58\right)$, and ball speed $\left(\;p<0.001,\omega =0.99,\eta_{p}^{2}=0.33\right)$. *Post-hoc* analysis highlighted that these dependent variables displayed different changes across the three successive swing sessions. The peak vGR of the lead foot increased from *t*_0_ to *t*_1_ (*p* < 0.001), but did not show further increments from *t*_1_ to *t*_2_ (Figure 1a); club speed increased from *t*_0_ to *t*_1_ (*p* < 0.001) and continued to increase, though to a lesser extent, from *t*_1_ to *t*_2_ (*p* = 0.002) (Figure 2a); ball speed did not change significantly from *t*_0_ to *t*_1_, but increased from *t*_1_ to *t*_2_ (*p* = 0.001) (Figure 2b).

**Figure 1:**
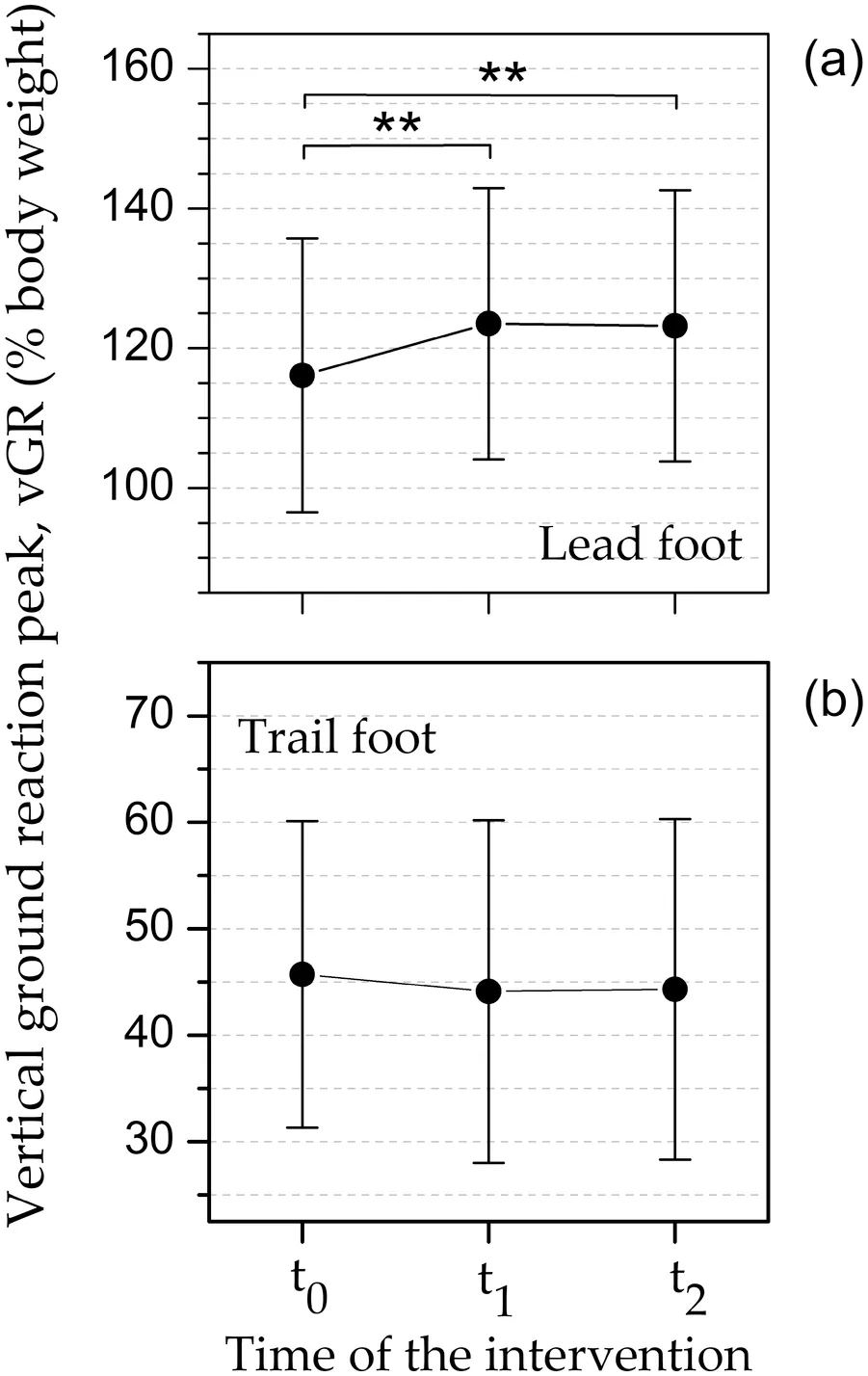
Peak vertical ground-reaction force (vGR) for the lead foot **(a)** and trail foot **(b)** recorded during three separate swing sessions: the first session before the foot proprioceptive intervention (time *t*_0_), the second soon after the intervention (time *t*_1_), and the third three minutes after the end of the second session (time *t*_2_). *p < 0.05; **p < 0.001.

**Figure 2:**
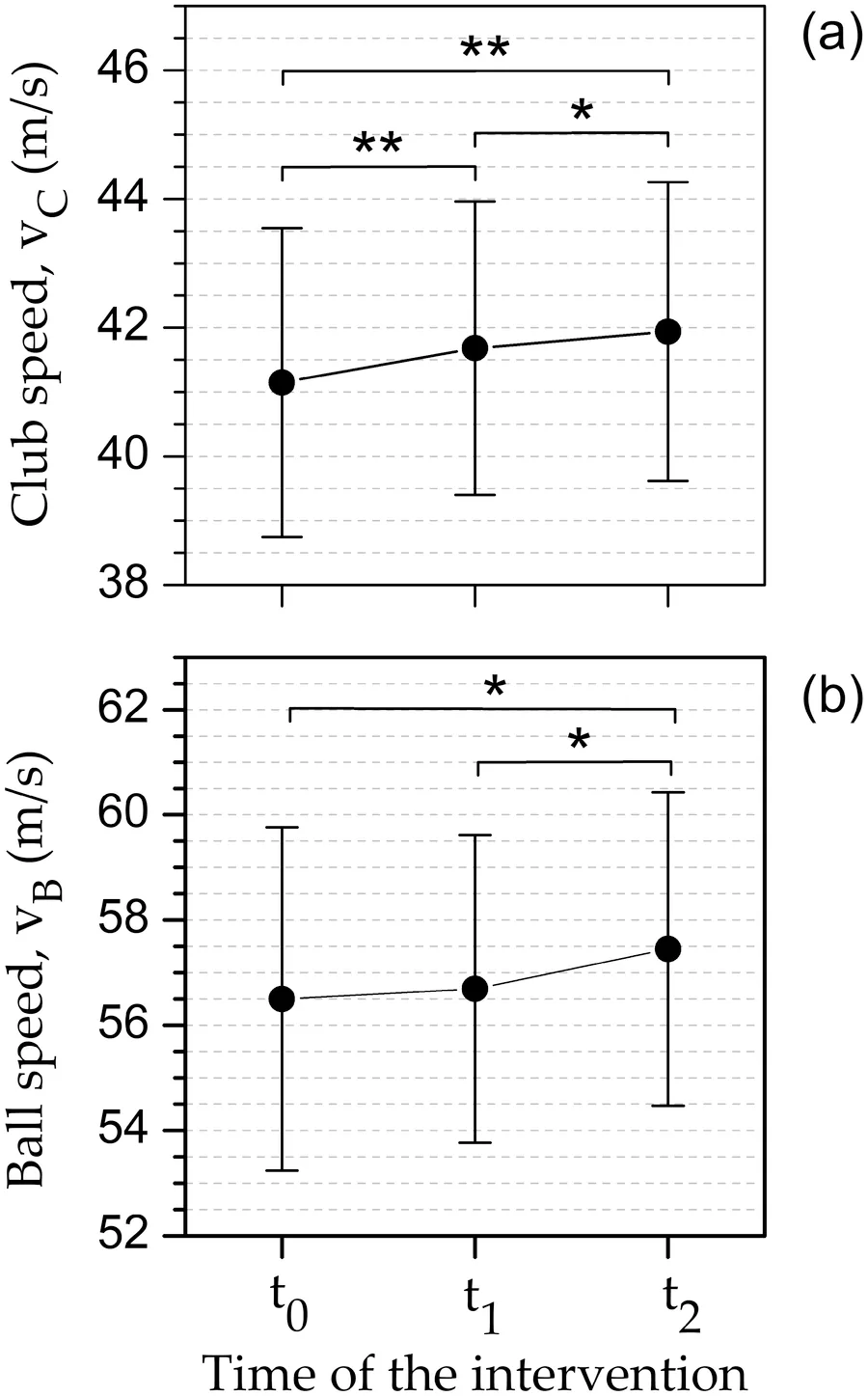
Club speed $v_{C}$ **(a)** and ball speed $v_{B}$ **(b)** recorded during three separate swing sessions: the first session before the foot proprioceptive intervention (time *t*_0_), the second soon after the intervention (time *t*_1_), and the third three minutes after the end of the second session (time *t*_2_). *p < 0.05; **p < 0.001.

In contrast to the lead foot, trail-foot peak vGR did not differ significantly across the three assessment times (Figure 1b). The phase of the swing movement at which the peak vGR of the lead foot occurs was also not affected by the treatment. This peak occurred shortly before phase P6 of the swing action (see appendix).

Figures 3 and 4, along with Table 2, display the outcomes of the one-sample *t*-test performed on the three sets of data obtained by calculating the individual differences between the performances at two different stages of the treatment for each athlete and then averaging these differences across participants. These analyses provided complementary estimates of the direction and magnitude of the within-subject changes and were not used as the primary basis for statistical inference.

**Figure 3:**
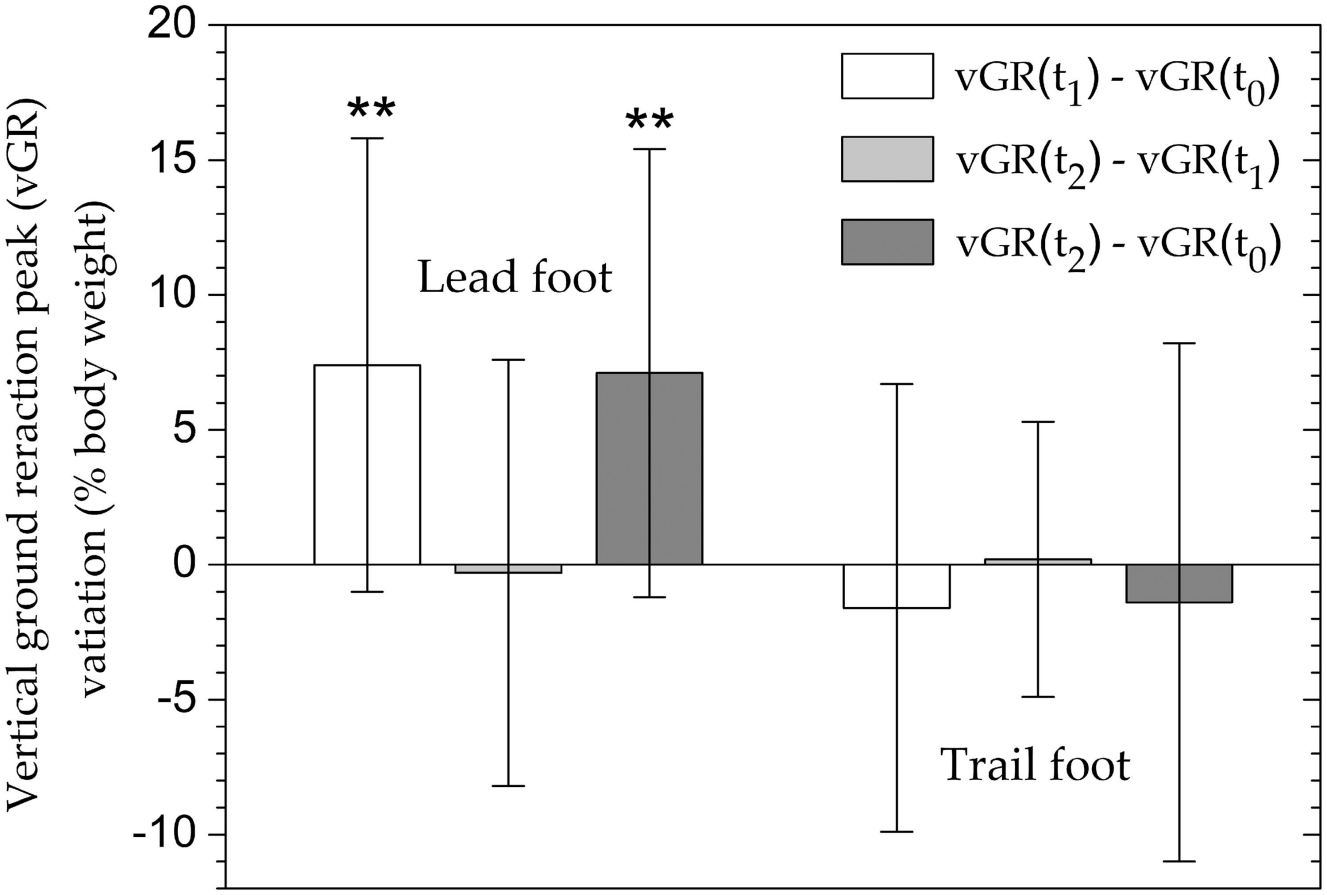
Differences in peak vertical ground-reaction force (vGR) for the lead foot and trail foot between each pair of three swing sessions: the first session before the foot proprioceptive intervention (time *t*_0_), the second soon after the intervention (time *t*_1_), and the third three minutes after the end of the second session (time *t*_2_). The differences were calculated by taking the individual differences between the performances in two sessions for each athlete and then averaging these differences across participants. Mean ≠ 0: *p < 0.05; **p < 0.001.

**Figure 4:**
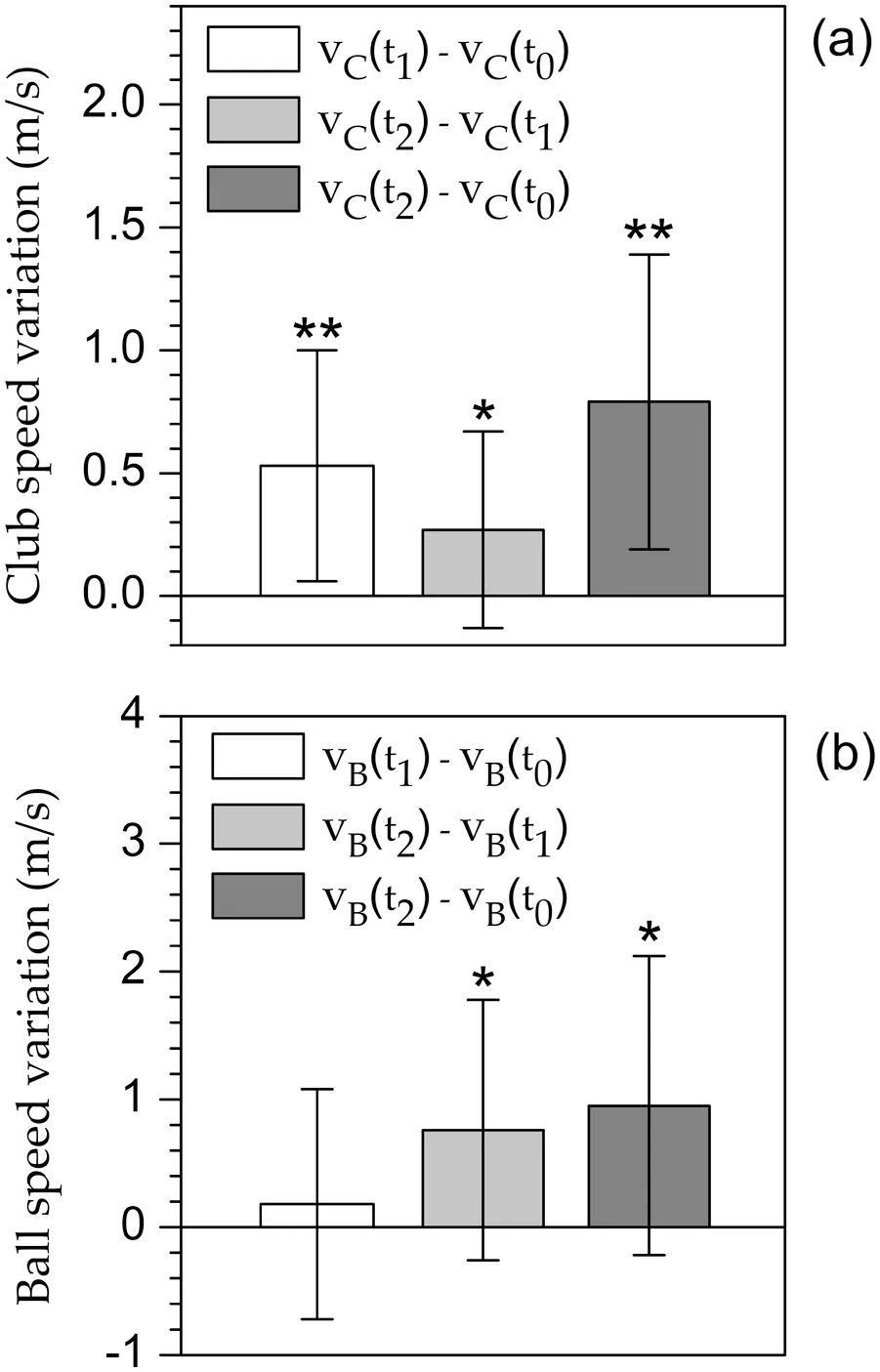
Differences in club speed **(a)** and ball speed **(b)** between each pair of three swing sessions: the first session before the foot proprioceptive intervention (time *t*_0_), the second soon after the intervention (time *t*_1_), and the third three minutes after the end of the second session (time *t*_2_). The differences were calculated by taking the individual differences between the performances in two sessions for each athlete and then averaging these differences across participants. Mean ≠ 0: *p < 0.05; **p < 0.001.

**Table 2:** One sample *t*-test outcomes (null hypothesis mean = 0, alternative hypothesis mean ≠ 0) for the individual differences between the performances at two different stages of the treatment for each athlete averaged over the participants [BW = body weight, vGR = vertical ground-reaction force, *d* = Cohen's effect size, with *d* = 0.2 indicating a small effect, *d* = 0.5 a medium effect, and *d* = 0.8 a large effect (35)].

Individual performance difference at two stages of the treatment	mean ± SD	*d*	*p*	Power	Confidence interval	
					Lower limit	Upper limit
Difference from *t*_1_ to *t*_0_						
Lead foot vGR (% BW)	7.4 ± 8.4	0.88	<0.001	0.99	3.9	10.8
Club speed (m/s)	0.53 ± 0.47	1.13	<0.001	>0.99	0.33	0.72
Ball speed (m/s)	0.18 ± 0.90	0.20	0.31	0.17	−0.18	0.55
Difference from *t*_2_ to *t*_1_						
Lead foot vGR (% BW)	−0.3 ± 7.8	0.04	0.84	0.05	−3.6	2.9
Club speed (m/s)	0.27 ± 0.40	0.67	0.003	0.90	0.10	0.43
Ball speed (m/s)	0.76 ± 1.02	0.74	0.001	0.95	0.34	1.19
Difference from *t*_2_ to *t*_0_						
Lead foot vGR (% BW)	7.1 ± 8.3	0.86	<0.001	0.98	3.6	10.5
Club speed (m/s)	0.79 ± 0.60	1.31	<0.001	>0.99	0.57	1.04
Ball speed (m/s)	0.95 ± 1.17	0.81	0.001	0.97	0.47	1.43

## Discussion

The present study showed distinct acute temporal changes in lead-foot peak vGR, club speed, and ball speed following the foot-focused conditioning intervention and modified swing instructions. Lead-foot peak vGR increased immediately after the intervention and remained essentially unchanged thereafter, whereas club speed increased at *t*_1_ and further increased at *t*_2_. In contrast, ball speed increased significantly only at *t*_2_. This temporal sequence is consistent with a progressive short-term adjustment to the modified swing pattern.

At time *t*_1_, immediately after the intervention, participants appeared to use their feet differently during the swing compared with time *t*_0_, as reflected by a higher lead-foot peak vGR. This increase was accompanied by higher club speed, but not by a significant change in ball speed. Ball speed is mainly (about 75%) determined by clubhead speed; however, centeredness of impact also has a considerable effect (about 7%) on ball speed (39). One possible interpretation is that integrating the new foot technique, although accompanied by higher club speed, may initially have challenged impact control and precision because it differed from the motor pattern consolidated over years of practice. However, impact control and precision were not directly assessed.

At time *t*_2_, three minutes after the intervention, participants continued to use their feet as they did at time *t*_1_ (at least regarding the peak vGR of the lead foot), but they transferred even more speed to the club (greater than it was at time *t*_1_) and finally managed to increase the ball speed after impact. In the second swing session after the conditioning intervention, and after three minutes of focusing on the sensations provided by the new technique, participants may have progressively integrated and harmonized the new foot motor pattern within the swing gesture, which may have contributed to the subsequent increase in ball speed. Ultimately, the pattern of observed changes following the intervention and the modified swing technique appeared to progressively propagate, with practice, from the feet to the club and finally to the ball. Any interpretation in terms of proprioceptive processing, neuromuscular control, sensorimotor integration, or motor-pattern learning remains hypothetical, as these variables were not directly assessed.

The increase in lead-foot peak vGR observed in the present study is consistent with previous evidence highlighting the importance of golfer–ground interaction for swing performance. Han et al. (32) reported a strong association between lead-foot peak vGR and maximum clubhead speed in skilled male golfers, supporting the relevance of the vertical ground-reaction-force component observed in the present study. More broadly, Faux et al. (33) showed that experimentally altering foot center of pressure can modify lower-body kinematics during the golf swing, while Watson et al. (34) identified ground-reaction-force and center-of-pressure variables as relevant biomechanical factors associated with clubhead speed and skill level. Thus, the present findings are broadly consistent with previous evidence supporting the importance of foot–ground interaction in golf performance. However, direct comparison is limited because these studies did not examine the same structured foot-conditioning intervention or the acute temporal progression observed here. The present results therefore extend, rather than directly replicate, previous findings by suggesting that targeted foot-action training and its repeated application during the swing may be accompanied by short-term changes in both foot loading and swing-performance variables.

From *t*_0_ to *t*_2_ mean club speed increased by 0.79 m/s and mean ball speed by 0.95 m/s, corresponding to large standardized effects (*d* = 1.31 and *d* = 0.81, respectively). The increase in ball speed represented an improvement of approximately 1.7%. Although modest in absolute terms, this change may have practical relevance because it was observed in highly skilled golfers with very low handicaps and extensive training experience, in whom the scope for further improvement may be comparatively limited. To our knowledge, however, the literature has not yet established a minimal worthwhile-change threshold specifically for 6-iron club speed or ball speed in highly skilled golfers. Consequently, it cannot be determined whether the observed increases exceed a formally validated threshold of practical meaningfulness. The persistence and competitive relevance of these acute changes should therefore be examined in future controlled studies.

Because the final assessment was conducted within the same testing session, only a few minutes after the intervention, the findings reflect exclusively acute responses and provide no evidence regarding retention, sustained effectiveness, or long-term training adaptations.

An important limitation of the study relates to the selection of participants (Table 1) and the limited duration of the proprioceptive conditioning intervention. Because the sample consisted exclusively of highly skilled male golfers with very low handicaps, the findings cannot be generalized to recreational or novice golfers, female athletes, junior golfers, or older populations. The participants all demonstrated an excellent perception of the quality of movements performed in the various phases of the swing, and fully perceived the variations implemented in the swing and their effects. Most reported experiencing greater freedom and fluidity of movement, with fewer constraints and less resistance. However, novice and less experienced players may not yet have developed the same level of kinesthetic awareness or movement quality as more seasoned golfers, which could impact the effectiveness of the conditioning intervention. Therefore, future studies should include male and female golfers with varying levels of experience, age, and skill, including recreational, novice, junior, and older golfers, as well as other factors that could influence the outcomes of proprioceptive conditioning. Furthermore, interventions of different durations could be tested to assess their effectiveness across diverse groups of golfers. Such an approach may enhance the generalizability of the findings, providing a more comprehensive understanding of the impact of the proprioceptive intervention and helping to tailor interventions more effectively for diverse player profiles. The age range of 18–65 years represented an eligibility criterion rather than the observed age distribution of the sample, whose mean age was 29 ± 10 years. Although participants were relatively homogeneous in terms of competitive experience, weekly practice, and handicap, this golf-specific homogeneity does not exclude possible age-related differences in proprioceptive function, balance, neuromuscular control, and acute motor adaptation. Because age was not examined as a moderator or covariate, its potential confounding influence cannot be excluded. Future studies should include larger age-stratified samples to determine whether acute responses to the intervention differ with age.

Another limitation arises from the limited number of parameters analyzed (peak vGR of the lead foot and the trail foot, club head speed, and ball speed) despite the large number of parameters recorded by the Trackman and the Smar*t2*Move Dual Force Plate systems during the three swing sessions. The selection of ball and club head speed among the Trackman parameters was justified by the following considerations: (1) ball speed is a key indicator of both swing speed and the quality of impact, making it fundamental to overall performance, and (2) club head speed is largely the primary determinant of ball speed (37, 39). Additionally, these parameters are less susceptible to variations from external environmental factors. For instance, ball flight data can be heavily influenced by changes in wind speed and direction, which can differ significantly between sessions. Han et al. (32) highlighted that among the parameters measured by force plate systems, the peak vGR values of the lead and trail feet are key factors for optimal swing mechanics. In particular, the peak vGR of the lead foot has been shown to correlate strongly with maximum clubhead speed, with the vertical component providing the dominant contribution (32).

During the measurement sessions, a 6-iron, which is an intermediate and frequently used club, was intentionally utilized. This should reasonably ensure good generalizability of the obtained results to other types of clubs. However, this aspect should also be confirmed with further investigations.

A further limitation concerns the force-plate data. The force plates used can only detect the vertical component of the ground reaction force and have indeed revealed an increase in the peak vGR of the lead foot with the modified swing technique. This technique involves intended forefoot-pressing and horizontal shear actions performed rhythmically with the right and left feet. Therefore, the use of triaxial platforms would likely have also detected an increase in the horizontal components of the ground reaction force and its axial moment on the plane of the platform. However, the monoaxial platforms used, due to their small size and portability, are particularly suitable for studying the typical golf actions, avoiding potential interference with the club during the swing or even visual distractions that could influence the player's movement. Furthermore, although participants were instructed to integrate the practiced foot-action sequence into the subsequent swings, the extent to which the complete sequence was reproduced during each swing was not independently quantified. Therefore, the observed increase in lead-foot peak vGR provides evidence of a change in foot loading during the swing but cannot be considered direct confirmation of the prescribed horizontal shear actions or of complete adherence to the foot-action sequence.

An important limitation of this study is the absence of a control, placebo, or sham condition. Although the methodological precautions adopted reduce the likelihood that the observed changes were solely due to practice or familiarization effects, the lack of a comparator limits the strength of causal inference and does not allow alternative explanations to be completely excluded. Several methodological precautions, however, were implemented to minimize this risk. First, the participants were all experienced golfers with prior familiarity with launch monitor assessments. This ensured that they were already accustomed to repeating swing shots under standardized testing conditions and that performance variability due to unfamiliarity with the equipment or procedures was minimized. Second, before the first testing session, all golfers completed their usual pre-competition warm-up routine and performed additional monitored swings until they reported having reached stable and representative ball speed values. This procedure helped establish a consistent baseline. Crucially, a complementary ANOVA conducted on the five swing shots within each session revealed no significant systematic trend across consecutive shots in any of the dependent variables. In particular, no significant changes were found in lead foot peak vGR (*p* = 0.54), club speed (*p* = 0.48), or ball speed (*p* = 0.57) across the five swings executed with the traditional technique during the first session at time *t*_0_. This finding, together with the absence of systematic trends during the baseline swings, supports the interpretation that the observed progression was unlikely to be explained solely by a simple within-session practice effect. Nevertheless, practice, short-term learning, warm-up, biological variability, and other time-related factors cannot be completely excluded in the absence of a comparator condition. As an exploratory investigation, the present study provides preliminary evidence of the feasibility and potential effectiveness of the proposed paradigm and supports further research with larger samples and controlled designs to verify the intervention-specific nature and persistence of the observed changes.

Further studies are also necessary to assess the impact of the new swing technique on the mechanical loading acting across the kinetic chain involved in the swing movement. However, this requires advanced biomechanical modelling designed to calculate the shear and axial joint reaction forces acting on the multi-joint kinetic chain (40) during high-velocity movements (41). This information is fundamental given the reported incidence of overuse injuries among golf players (42).

## Conclusion

In this study, we devised and administered to experienced golfers a targeted conditioning intervention based on a rhythmic sequence of ground actions exerted by the rearfoot and forefoot of both the lead and trail feet. Participants were then guided in modifying their swing technique to integrate the motor pattern acquired during the conditioning phase. This approach was followed by measurable acute changes in the selected performance-related swing parameters: lead-foot peak vGR, club speed, and ball speed. Notably, the observed changes following the intervention and the modified swing technique appeared to progressively propagate with practice from the feet to the club and ultimately to the ball. These acute findings suggest that the proposed training method may facilitate a progressive refinement of swing technique through greater integration of foot mechanics, with potential benefits for swing performance. These findings are specific to highly skilled male golfers with very low handicaps and require confirmation in other golfer populations.

## Data Availability

The raw data supporting the conclusions of this article will be made available by the authors, without undue reservation.

## Appendix A: The seven reference positions of the Smar*t2*Move Dual Force Plate system

The Smar*t2*Move Dual Force Plate system includes proprietary data-analysis software that identifies seven reference positions (P1–P7) during the golf swing:

P1: starting position

P2: club parallel to the ground in the backswing

P3: left arm parallel to the ground in the backswing

P4: apex of backswing

P5: left arm parallel to the ground in the downswing

P6: club parallel to the ground in the downswing

P7: impact of the club with the ball

In the Supplementary Material, Figures 5–12 display a sample of the Smar*t2*Move output parameters for each of the seven reference positions and in the position (between P5 and P6) where the peak vertical ground-reaction force occurs.
