# Supplementary material for: Training the proprioceptive and propulsive function of the foot for improved technique and enhanced performance of the golf swing: a pilot study
Source: Front Sports Act Living. 2026 Sep 14;8:1888684. doi: 10.3389/fspor.2026.1888684 (PMC13616624; doi:10.3389/fspor.2026.1888684)
Supplement: Supplementary file 1 [file Datasheet1.docx]

**Supplementary material**

Figure 5 to Figure 12 display a sample of the Smart2Move output parameters for each of the seven reference positions (P1: starting position; P2: club parallel to the ground in the backswing; P3: left arm parallel to the ground in the backswing; P4: apex of backswing; P5: left arm parallel to the ground in the downswing; P6: club parallel to the ground in the downswing; P7: impact of the club with the ball) and in the position (between P5 and P6) where the peak value of the vertical ground reaction occurs.

**Figure 5.** The first reference position of the Smart2Move Dual Force Plate system (P1): starting position.

**Figure 6.** The second reference position of the Smart2Move Dual Force Plate system (P2): club parallel to the ground in the backswing.

**Figure 7.** The third reference position of the Smart2Move Dual Force Plate system (P3): left arm parallel to the ground in the backswing.

**Figure 8.** The fourth reference position of the Smart2Move Dual Force Plate system (P4): apex of backswing.

**Figure 9.** The fifth reference position of the Smart2Move Dual Force Plate system (P5): left arm parallel to the ground in the downswing.

**Figure 10.** Position where the peak value of the vertical ground reaction occurs.

**Figure 11.** The sixth reference position of the Smart2Move Dual Force Plate system (P6): club parallel to the ground in the downswing.

**Figure 12.** The seventh reference position of the Smart2Move Dual Force Plate system (P7): impact of the club with the ball.
